# Nutritional Profiles and Factors Associated with the Intake of Certain Food Types in Patients Undergoing Maxillofacial Prosthetic Rehabilitation: A Cross-Sectional Study

**DOI:** 10.3390/dj13010029

**Published:** 2025-01-13

**Authors:** Nehasha Pradhan, Mai Murase, Masako Akiyama, Hiroko Tani, Yuka I. Sumita, Noriyuki Wakabayashi

**Affiliations:** 1 Department of Advanced Prosthodontics, Division of Oral Health Sciences, Graduate School of Medical and Dental Sciences, Institute of Science Tokyo, Tokyo 1138510, Japan; prad.mfp@tmd.ac.jp (N.P.); sumita@tky.ndu.ac.jp (Y.I.S.); wakabayashi.rpro@tmd.ac.jp (N.W.); 2 Young Investigator Support Center, Institute of Science Tokyo, Tokyo 1138510, Japan; m-akiyama.cell@tmd.ac.jp; 3 Maxillofacial Prosthetics Clinic, Institute of Science Tokyo Hospital, Tokyo 1138510, Japan; tani.mfp@tmd.ac.jp; 4 Department of Partial and Complete Denture, School of Life Dentistry at Tokyo, The Nippon Dental University, Tokyo 1028159, Japan

**Keywords:** head and neck cancer, maxillofacial prosthetics, nutritional intake, dysphagia, quality of life, dietary habits

## Abstract

**Background:** Malnutrition is a significant concern for head and neck cancer (HNC) patients, as treatment often impairs mastication, causes dysphagia, and alters taste and smell, leading to reduced food intake and a diminished quality of life. Thus, this study aims to compare nutritional intake in HNC survivors using maxillofacial prostheses (MFPs) to healthy reference values and identify the factors influencing their dietary intake. **Methods:** The study included 56 patients treated for HNC undergoing rehabilitation with comfortable definitive dentures for over a month at the Maxillofacial Prosthetics Clinic of Tokyo Medical and Dental University Hospital. Data were gathered on the demographics, clinical characteristics, malnutrition risk using a malnutrition universal screening tool, dietary intake consistency via a functional oral intake scale, swallowing difficulties with eating assessment tool-10, and nutrient intake through a Brief-type Self-administered Dietary History Questionnaire. Patients’ nutrient intakes were compared to the reference values from the BDHQ ad hoc computer algorithm based on the 2015 National Health and Nutrition Survey in Japan. Factors such as maximum mouth opening and the number of functional teeth were also assessed together with the aforementioned factors. **Results:** There were significant differences between the patient values and reference values, with lower intakes of total dietary fiber, carbohydrates, and β-carotene, while higher intakes of calcium, fats, and certain vitamins were noted in the patients. Food intake consistency, swallowing difficulties, and mouth opening significantly influenced green vegetable intake, whereas sex and the number of functional teeth impacted cereal intake. **Conclusions:** The HNC survivors were rehabilitated with MFP; however, their nutritional intake differed from that of healthy subjects. The significance of swallowing rehabilitation, appropriate food preparation, exercises to enhance mouth opening, and the preservation of functional teeth has been emphasized as critical factors influencing diet in head and neck cancer (HNC) survivors. Additionally, the importance of a multidisciplinary approach to nutritional care for these individuals is underscored.

## 1. Introduction

Malnutrition is a significant concern in oncology, especially for patients receiving rehabilitation after head and neck cancer (HNC) surgery [1]. According to the European Society for Clinical Nutrition and Metabolism guideline, screening for malnutrition is crucial [2]. Nutrition significantly aids recovery in cancer survivors [3]. HNC treatment often affects mastication and causes dysphagia [4], along with changes in taste and smell [5,6,7], resulting in decreased food intake and diminished quality of life. Caburet et al. found that adequate nutrition aids healing and minimizes postoperative complications [8]. The sensory changes and oral symptoms caused by such treatments affect food intake [9,10,11].

Maxillofacial prosthetic (MFP) rehabilitation plays a crucial role in restoring both form and function for patients who have undergone treatment for HNC and have lost portions of their oral or facial structures. This approach significantly improves their quality of life [12,13,14]. Systemic cancers, particularly NHC and gastrointestinal cancers, are associated with a decline in nutritional status, making it essential to use MFPs that support oral intake [15]. Despite the use of MFP, Morimata et al. noted that patients continue to face challenges related to quality of life. They suggested that prosthetic solutions alone might not be sufficient to fully address issues related to nutrition [16]. Tani et al. observed decreased body weight and resting energy expenditure at hospital discharge, linking Mini-Nutritional Assessment-Short Form (MNA-SF) scores and C-reactive protein to energy expenditure [17]. Yanagi et al. found that over half of the patients undergoing MFP rehabilitation were malnourished at the time of diagnosis, and that the duration of MFP uses and a history of neck dissection significantly impacted the MNA-SF scores [18]. Their study conducted a general screening for malnutrition; however, it did not investigate details of nutrients. Therefore, the study provided limited information to patients and treating prosthodontists. A more detailed investigation of the nutritional profile of these patients, including specific nutrients, is required to gain a better understanding and improve patient care. A varied diet rich in nutrient-dense foods is essential for meeting these patients’ heightened nutritional needs [19]. Understanding food types helps patients reflect on their eating habits [3,20], and a study found that multiple factors affected dietary intake after surgical interventions [21]. Several studies have looked at prostheses’ impact on dietary intake, especially fruits and vegetables, in the elderly, yielding positive [22,23,24] and negative [25] results.

The utilization of non-invasive investigative tools that impose minimal burden on the patient, such as the Brief-type Self-administered Dietary History Questionnaire (BDHQ), can prove advantageous in the examination of nutritional status and dietary intake [26,27,28,29]. Multiple studies on cancer patients have selected a validated tool that was easy and simple to screen patients’ nutritional risk, the malnutrition universal screening tool (MUST). It has shown its strength for application with adult patients across all healthcare settings, including oncology [30,31]. The eating assessment tool (EAT-10) allows quick and easy dysphagia assessment and has been used successfully in assessing swallowing disabilities in HNC patients [32,33]. The Food Oral Intake Scale (FOIS) is a useful tool that helps us understand patients’ oral intake habits commonly used in cancer patients [34]. Matsuda et al. evaluated self-efficacy and dysphagia in cancer patients using the Food Oral Intake Scale (FOIS), noting changes in food consistency [35]. Few studies explored the association between the remaining number of functional teeth and eating habits, dietary intake, and MFP stability and found negative and positive relationships [36,37]. However, the data on the relationship between this and food intake is still minimal. Several studies have investigated the link between tooth loss and nutritional status, finding an association between malnutrition and tooth loss in elderly individuals [38]. In patients treated for HNC, trismus is a common complication [39,40], which can affect both the quantity and consistency of food intake. This issue warrants further exploration.

This study aims to (1) compare the nutritional intake variation in HNC survivors undergoing rehabilitation with MFP to healthy reference values, providing insights into the dietary profile of this patient group for better treatment planning, and (2) identify factors influencing food intake in these patients to better understand their dietary needs and guide with multidisciplinary supportive care strategies.

## 2. Materials and Methods

### 2.1. Subjects

Outpatients receiving rehabilitation treatment after HNC surgery at the Maxillofacial Prosthetics Clinic of Tokyo Medical and Dental University Hospital were recruited. A retrospective review of the medical records was performed to collect details, including age, sex, treatment site, neck dissection history, and chemotherapy/radiotherapy history, and the participants needed to complete questionnaires and have their weight and height measured. Inclusion criteria required the following: (i) patients who have undergone head and neck cancer treatment, and (ii) have used a stable, comfortable, definitive maxillofacial prosthesis for over a month. Exclusion criteria were as follows: (i) inability to communicate in Japanese, (ii) being under dietary counseling, (iii) reliance on tube feeding, and (iv) suffering from any metabolic disorder such as recurrent carcinoma, endocrine disease, and diabetes mellitus. Originally, 75 participants were recruited over three months (September to December 2022), with 19 excluded due to incomplete data. This study was performed in line with the principles of the Declaration of Helsinki. The ethics approval was granted by the Tokyo Medical and Dental University ethics committee (approval number D2014-076-07, date of approval: 8 September 2014–31 March 2027), and written informed consent was obtained from all the participants.

### 2.2. Methods

Height and weight were measured using an A&D machine (Tokyo, Japan). Malnutrition risk was assessed with the malnutrition universal screening tool (MUST), and nutrition was evaluated using the Brief-type Self-administered Dietary History Questionnaire (BDHQ). Swallowing function was assessed with the eating assessment tool-10 (EAT-10), and dietary consistency and habits were evaluated using the functional oral intake scale (FOIS).

#### 2.2.1. Overall Screening for Malnutrition

Malnutrition risk was screened with the MUST, which is a five-step globally recognized malnutrition screening tool for adults, which takes into account body mass index (BMI) (0, BMI > 20; 1, BMI 18.5–20.0; 2, BMI < 18.5), history of unplanned weight loss in the previous 3–6 months (0 if weight loss is <5%; 1 if weight loss is 5–10%; 2 if weight loss is >10%), and presence of acute illness or inability to eat for more than 5 days (2 points if either is applicable). The risk of malnutrition is obtained by summing all three scores (0, low; 1, medium; 2, high).

#### 2.2.2. Eating Assessment Tool-10

The EAT-10 evaluates swallowing difficulties. It has ten questions addressing issues such as weight loss, meal participation, effort, pain, throat obstruction, coughing, and stress during eating. Responses are scored from 0 (no problem) to 4 (severe problem), with a total score of ≥3 indicating potential swallowing issues.

#### 2.2.3. Functional Oral Intake Scale

The FOIS assesses the ability to swallow food according to its consistency. It has a 7-point scoring system (1, no oral feeding; 2 and 3, tube-dependent feeding; 4, total oral intake of single-consistency food; 5, total oral intake of specially prepared multiple-consistency food; 6, total oral intake with no special preparation but avoidance of specific foods or liquids; and 7, total oral intake with no restrictions). Only the patients who were on oral intake were included.

#### 2.2.4. Assessment of Nutritional and Dietary Food Intake

The BDHQ is a 4-page questionnaire with 80 items on food intake, frequency, dietary habits, and cooking methods over the past month. It calculates the daily intake of 16 key nutrients [calcium, iron, vitamin C, total dietary fiber (TDF), potassium, sodium, fats, saturated fatty acid (SFA), cholesterol, folic acid, total energy, protein, carbohydrate, magnesium, b carotene equivalent (β-carotene), and vitamin D] and six food types common in Japanese cuisine [i.e., meat, fish, green vegetables, other vegetables, fruits, and cereals]. Data from these patients were inserted in an online BDHQ ad hoc computer algorithm which provides the analysis of all the data and provides healthy reference values, categorized by age and sex, which is based on the 2015 National Health and Nutrition Survey in Japan [41]. Additionally, factors influencing the types of food intake were analyzed [28].

#### 2.2.5. Measurement of Maximum Mouth Opening

**Figure 1:**
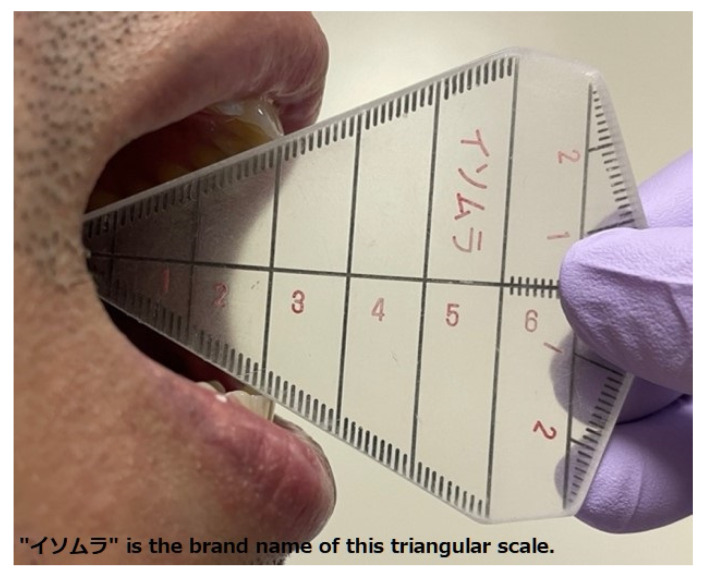
The measurement of the maximum mouth opening using a triangular scale (Isomura).

The conventional triangular scale was used to measure the maximum mouth opening between the incisal edges of natural or artificial central incisors in the maxillary and mandibular arches while wearing the denture (Figure 1).

#### 2.2.6. The Number of Functional Teeth Remaining

The number of remaining functional teeth was recorded considering functionally sound crowns. Endodontically treated teeth with healthy crowns were included.

### 2.3. Statistical Analysis

The values are shown as the mean ± (standard deviation), median, and quartiles (Q1–Q3). All the statistical analyses were performed using SPSS for Windows (version 26.0; IBM Corporation, Tokyo, Japan). A two-sided *p*-value of <0.05 was considered statistically significant.

#### 2.3.1. Nutritional Intake vs. Reference Value

**Figure 2:**
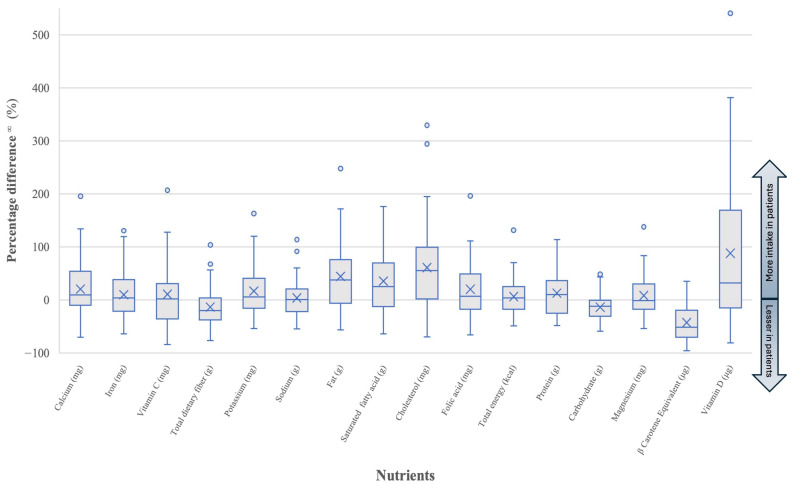
Percentage difference in intake of 16 nutrients between the study group and a healthy reference value. For the box-and-whisker plot, the line in the middle indicates the median, and “×” marks show the mean values. The top and bottom lines show the first and third quartiles and the whiskers extend to 1.5 times the height of the box or, if no case/row has a value in that range, to the minimum or maximum values. Data points that fall outside the whiskers are plotted as individual dots. ^∞^ percentage difference = (intake value of patients − reference value)/reference value × 100. Abbreviations: g—gram; mg—milligram; μg—microgram; kcal—Kilocalories.

The percentage difference in 16 nutrient intakes between the patients and the reference value from the BDHQ algorithm was calculated as (intake value of patients − reference value)/reference value × 100. The graph in Figure 2 did not indicate a normal distribution, so the comparison was performed using the Wilcoxon signed-rank test.

#### 2.3.2. Factors Influencing Types of Food Intake

Factors affecting the intake of the six food types included in the BDHQ were analyzed using multiple linear regression analysis with the intake of food types such as green vegetables, other vegetables, meat, fish, fruits, and cereals as the dependent variables. The independent variables were sex, FOIS score, EAT-10 score, MUST score, maximum mouth opening, and number of functional teeth. Sex was categorized as male (1) or female (2); the FOIS score as 4, 5, 6, or 7; the EAT-10 score as 1 (without swallowing disability) or 2 (with swallowing disability); the MUST scores as 0 (no risk of malnutrition) or 1 (medium risk) or 2 (high risk); maximum mouth opening (mm); and the number of functional teeth (n). The maximum mouth opening and the number of functional teeth were measured as continuous variables.

## 3. Results

### 3.1. Demographic Data

Fifty-six eligible patients (mean age 73 ± 9 years; 32 women, 24 men) were included in this study. Their characteristics are summarized in Table 1. Among them, 24 patients had a maxillary defect, with 2 involving the pharynx (43%); 19 had a mandibular defect, including glossectomy (34%); and 9 had both maxillary and mandibular defects (16%). Four patients received only chemo/radiation therapy. Only 22 patients (39%) underwent neck dissection. Five patients (9%) were completely edentulous, while fifty-one (91%) were partially edentulous. Chemotherapy was administered to 29 patients (52%), and 34 (61%) had a history of radiotherapy. The risk of malnutrition was classified as medium in 11 patients (20%) and considerable in 15 (27%). According to the EAT-10, 30 patients (54%) experienced swallowing impairment. The FOIS results indicated that 3 patients (5%) could eat single-consistency food, 15 (27%) could eat specially prepared multiple-consistency food, and 12 (21%) could eat without special preparation but avoided certain foods.

### 3.2. Nutritional Intake Compared with Reference Value

The comparison of nutrient intake between the patients and the reference value (Table 2) showed significant differences in calcium, total dietary fiber (TDF), fats, saturated fatty acid (SFA), cholesterol, folic acid, carbohydrates, β-carotene, and vitamin D (*p* < 0.05, Wilcoxon signed-rank test). However, no significant differences were observed in iron, vitamin C, potassium, sodium, magnesium, protein, or total energy intake (*p* > 0.05).

Figure 2 illustrates the percentage differences in nutrient intake. Most patients had lower intakes of TDF, vitamin C, carbohydrates, and β-carotene, while calcium, potassium, fats, SFA, cholesterol, folic acid, magnesium, and vitamin D were higher.

### 3.3. Factors Influencing Type of Food Intake

Table 3 presents the intake values for six food types commonly found in Japanese cuisine. The results of the multiple regression analysis identifying the factors affecting the intake of green vegetables and cereals are shown in Table 4. The variance inflation factor indicated no multicollinearity concerns, ranging from 1.10 to 1.48.

For green vegetable intake, the FOIS (β = −0.40, *p* = 0.010), EAT-10 scores (β = −0.36, *p* = 0.015), and maximum mouth opening (β = 0.34, *p* = 0.013) were independently associated. Cereal intake was significantly influenced by patient sex (β = −0.27, *p* = 0.045) and the number of functional teeth (β = 0.30, *p* = 0.025).

The R^2^ and adjusted R^2^ values were 0.12 (0.01) for the intake of meat, 0.06 (−0.06) for fish, 0.16 (0.06) for other vegetables, and 0.02 (−0.1) for fruits. None of the factors showed any significant association with the intake of these food items.

## 4. Discussion

The results from the MUST indicated that nearly half (26 patients, 46%) of the HNC survivors undergoing MFP rehabilitation were at risk of malnutrition or already malnourished. This aligns with previous findings using the MNA-SF [17]. Dietary counseling often stops after hospital discharge, and this high percentage emphasizes the need for ongoing nutritional assessment during rehabilitation. According to the EAT-10, 26 of the 56 patients (46%) still experienced impaired swallowing, complicating regular food intake. The remaining 54% may not have reported difficulties due to oral rehabilitation appliances, such as definitive obturators and palatal augmentation prostheses, which enhance dietary intake [27]. The FOIS results indicated that 54% (30 patients) of the patients consumed specially prepared single-consistency foods, or avoided certain items, likely contributing to their differing nutrient intake compared to the reference values.

The comparison of nutrient intake between the HNC survivors with MFP and reference values revealed significant differences in several key nutrients. This study indicated notable deficiencies in total dietary fiber, carbohydrates, and β-carotene among the participants compared to the healthy reference values. These findings are consistent with previous studies that have reported reduced fruit and vegetable consumption in cancer survivors, often attributed to sensory and functional impairments caused by treatment interventions such as radiotherapy and surgery [4,8,42]. Dietary fiber intake among the participants ranged from 13 ± 5 g/day, falling below the dietary goal for preventing lifestyle diseases (≥17 g/day) [41]. Low dietary fiber intake is also linked to a higher risk of common non-communicable diseases, including cardiovascular diseases, type 2 diabetes, and several cancers [43]. The decline in dietary fiber-rich food intake may result from challenges such as dysphagia, trismus, and alterations in taste and smell, as identified through the EAT-10 and FOIS assessments in this study. Although the participants’ β-carotene intake was lower, no minimum dietary goal was established for it, as β-carotene is one of several provitamin A carotenoids (vitamin A precursors). Similarly, while carbohydrate intake was below reference values, it still exceeded the minimum dietary goal, indicating no associated risks.

Despite these deficiencies, some nutrients, including calcium, fats, saturated fats, cholesterol, folic acid, and vitamin D, were consumed at levels exceeding the reference values. This discrepancy may reflect a reliance on processed, easily consumable foods or dairy products, which may be easier to chew and swallow but lack the nutrient diversity required for optimal health. Although the intakes of calcium, vitamin D, and folic acid were higher than the reference values, they remained within the tolerable upper intake levels of 2500 mg/day, 100 μg/day, and 900 μg/day, respectively [41]. The upper limit for cholesterol intake is not established; however, it is recommended to be less than 200 mg/day. In the participants, cholesterol intake ranged from 461 ± 220 mg/day, which exceeds the recommended level. For total fat, the dietary goal is 20–30 g/day, but this study found that the participant intakes ranged from 60 ± 24 g/day, surpassing the recommended levels. The overconsumption of these nutrients is associated with an increased risk of certain cancers, cardiovascular diseases, and obesity [44]. The World Health Organization recommends that saturated fat intake (SFA) should be less than 7% of the total energy intake, and the participants’ intakes were found to align with this recommendation. While the intakes of iron, vitamin C, sodium, total energy, and magnesium were like healthy reference values, this suggests that the patients prioritized balanced diets despite facing potential challenges and that it is also possible to consume nutrients by changing the form of the food.

This study identified that FOIS scores, EAT-10 scores, and maximum mouth opening significantly impacted green vegetable intake. Sex and the number of functional teeth affected cereal intake using multiple regression [17]. The number of functional teeth also played a critical role, with fewer teeth correlating with reduced dietary diversity, particularly in foods requiring mastication, such as green vegetables. This underscores the importance of oral health preservation and prosthesis optimization for maintaining adequate nutritional intake. Prior studies have also shown that tooth loss is closely linked to malnutrition, particularly in older adults [22,24,37]. However, other food types showed no such associations. This suggests that factors beyond masticatory ability, such as cooking skills and dietary preferences, also play crucial roles in determining food intake. As noted in previous reports [45], prosthetic measures alone are unlikely to improve nutritional intake. This is because masticatory ability and masticatory efficiency are not the only factors affecting food consumption. Therefore, a multifaceted approach is necessary to address nutritional issues effectively.

In summary, adopting a multidisciplinary approach in maxillofacial prosthodontics fosters a more integrated, patient-centered, and efficient healthcare system. Prosthetic rehabilitation is a long-term necessity for patients, ensuring they receive comprehensive and coordinated care that enhances health outcomes and quality of life. This study highlights the importance of such an approach in addressing the nutritional needs of HNC survivors by involving dietitians, dental hygienists, speech–language therapists, and clinical psychologists. In addition to MFP rehabilitation, tailored dietary interventions are vital. For instance, nutrient-dense and easily consumable options like fiber- and β-carotene-enriched smoothies or pureed diets can address specific deficiencies while accommodating the physical and functional challenges identified in this study. The use of validated dietary assessment tools, such as the BDHQ and FOIS, can guide these interventions by providing detailed insights into individual nutritional needs [35]. Additionally, routine malnutrition screening with tools like MUST should be integrated into the care pathway to identify at-risk patients early and enable timely interventions. Addressing modifiable factors such as swallowing function and trismus through targeted therapies, including physiotherapy and speech therapy, may further enhance dietary intake and the overall quality of life [16].

While this study provides valuable insights, it is not without limitations. The relatively small sample size and single-center design may limit the generalizability of the findings. The study’s cross-sectional retrospective design limits the ability to identify long-term changes [31]. To explore how poor nutritional status impacts treatment effectiveness, there is a need for more intervention studies in the future [46]. Additionally, the retrospective nature of the data collection and the reliance on self-reported dietary habits could introduce reporting biases and its exclusive focus on Japanese dietary practices may limit its applicability in other populations. The patient compliance and limitation of resources might also have potential barriers to implementing suggested interventions. The ad hoc BDHQ algorithm’s database still uses the 2015 National Health and Nutrition Survey, Japan. Although all the study subjects were using MFP rehabilitation of a certain kind, it is yet not confirmed whether the difference in nutrients and food intake was affected by this or not, so future research should explore how specific MFP designs, differences in cancer treatment methods, and degree of progression influence nutrient intake and assess the long-term effects of nutritional interventions in this vulnerable population. Longitudinal studies are needed to track food intake changes and investigate other factors affecting nutrition in patients undergoing MFP rehabilitation.

## 5. Conclusions

- The HNC survivors using an MFP exhibited notable differences in nutrient intake compared to the reference values for 9 of the 16 nutrients in this study, suggesting that there is still improvement needed to improve the nutrition status and this needs more in-depth longitudinal and intervention studies in the future.
- Factors such as swallowing difficulties, food consistency modification, and maximum mouth opening significantly influenced the intake of green vegetables, and these need to be addressed.
- Effective maxillofacial prosthetic rehabilitation requires dental interventions such as mouth opening training, residual tooth preservation, occlusion restoration, swallowing training, and the regular monitoring of nutritional status, along with dietary counseling to improve nutritional intake.

## Figures and Tables

**Table 1:** Characteristics of the patient population undergoing maxillofacial prosthetic rehabilitation after treatment for head and neck cancer.

Variable	Value
Age (years)	73 ± 9 73 (68–80)
Sex	
Male	24 (43%)
Female	32 (57%)
Primary surgery site	
Maxillary, pharyngeal	24 (43%)
Mandibular, tongue	19 (34%)
Maxillary and mandibular	9 (16%)
Not applicable	4 (7%)
Neck dissection	
Yes	22 (39%)
No	34 (61%)
Dentition status	
Completely edentulous	5 (9%)
Partially edentulous	51 (91%)
Chemotherapy	
Yes	29 (52%)
No	27 (48%)
Radiotherapy	
Yes	34 (61%)
No	22 (39%)
MUST score	
0 (no risk)	30 (54%)
1 (medium risk)	11 (20%)
>2 (high risk)	15 (27%)
EAT-10 score	
<3 (without disability)	26 (46%)
≥3 (with disability)	30 (54%)
FOIS score	
4	3 (5%)
5	15 (27%)
6	12 (21%)
7	26 (46%)
Maximum mouth opening (mm)	
≤15	4 (7%)
16–29	20 (36%)
30–34	12 (21%)
≥35	20 (36%)
The number of functional teeth (*n*)	16 ± 7 17 (14–21)

The values for the age and number of functional teeth are shown as the mean ± standard deviation, median, and quartiles (Q1–Q3), and others are represented as count and percentage. Abbreviations: EAT-10, Eating Assessment Test; FOIS, functional oral intake scale; MUST, malnutrition universal screening tool.

**Table 2:** Comparison of results for the intake of 16 nutrients between patients requiring MFP rehabilitation after head and neck cancer surgery and a healthy reference value.

Nutrients	Patients	Reference Value	*p*-Value
Calcium (mg)	626 ± 276	524	0.014 *
	582 (450–717)	535 (510–554)	
Iron (mg)	9 ± 4	8	0.219
	8 (7–11)	8 (8–9)	
Vitamin C (mg)	131 ± 72	120	0.639
	122 (79–160)	125 (113–125)	
TDF (g)	13 ± 5	15	0.001 *
	12 (9–16)	16 (14–16)	
Potassium (mg)	2814 ± 1114	2442	0.080
	2575 (2076–3283)	2482 (2340–2623)	
Sodium (g)	11 ± 4	11	0.688
	11 (9–13)	11 (10–12)	
Fats (g)	60 ± 24	43	≤0.001 *
	59 (44–73)	43 (38–49)	
SFA (g)	17 ± 7	13	≤0.001 *
	16 (12–20)	13 (11–14)	
Cholesterol (mg)	461 ± 220	290	≤0.001 *
	450 (309–584)	281 (260–315)	
Folic acid (mg)	397 ± 174	335	0.033 *
	367 (296–477)	388 (315–357)	
Total energy (kcal)	1852 ± 579	1759	0.443
	1775 (1470–2181)	1749 (1538–1980)	
Protein (g)	75 ± 27	67	0.091
	72 (54–90)	67 (61–75)	
Carbohydrate (g)	226 ± 68	264	≤0.001 *
	223 (186–270)	254 (242–294)	
Magnesium (mg)	277 ± 96	259	0.396
	264 (209–333)	262 (243–285)	
β Carotene (μg)	3925 ± 2334	6940	≤0.001 *
	3480 (2039–5348)	7243 (6447–7263)	
Vitamin D (μg)	17 ± 13	9	≤0.001 *
	12 (8–21)	9 (8–10)	

Data are shown as the mean ± standard deviation, median, and quartiles (Q1–Q3). * *p* < 0.05. Abbreviations: SFA, saturated fatty acid; TDF, total dietary fiber.

**Table 3:** Patient’s intake of six types of food widely used in Japanese cuisine.

Food Types	Patient Data
Total amount of meat (g/day)	62 ± 36
	59 (37–80)
Total amount of fish (g/day)	94 ± 68
	82 (45–114)
Green vegetables (g/day)	120 ± 89
	103 (61–154)
Other vegetables (g/day)	141 ± 82
	119 (86–182)
Fruits (g/day)	101 ± 62
	93 (55–137)
Cereal (g/day)	112 ± 70
	87 (64–151)

Data are shown as the mean ± standard deviation, median, and quartiles (Q1–Q3).

**Table 4:** Outcome of the multiple regression analysis of the factors potentially affecting the intake of green vegetables and cereals.

	Predictor	Unstandardized Partial Regression Coefficient			Beta	t-Value	*p*-Value	VIF	R^2^ (Adj R^2^)
		Estimate	SE	95% CI					
Green vegetables	(Constant)	262.18	89.68	81.95~442.40		2.923	0.005		0.25 (0.16)
	Sex	9.92	17.72	−25.70~45.53	0.07	0.559	0.578	1.14	
	FOIS	−27.62	10.31	−48.34~−6.90	−0.40	−2.679	0.010 *	1.48	
	EAT-10	−47.57	18.78	−85.30~−9.84	−0.36	−2.533	0.015 *	1.30	
	MMO	2.10	0.81	0.47~3.73	0.34	2.585	0.013 *	1.15	
	NFT	0.31	1.21	−2.12~2.74	0.03	0.253	0.801	1.11	
	MUST	4.08	10.65	−17.31~25.48	0.05	0.384	0.703	1.23	
Cereal	(Constant)	90.09	93.51	−97.83~278.02		0.963	0.340		0.25 (0.15)
	Sex	−38.07	18.48	−75.2~−0.93	−0.27	−2.06	0.045 *	1.14	
	FOIS	8.09	10.75	−13.52~29.69	0.11	0.752	0.456	1.48	
	EAT-10	2.74	19.58	−36.6~42.09	0.02	0.14	0.889	1.30	
	MMO	−0.33	0.85	−2.03~1.37	−0.05	−0.391	0.697	1.15	
	NFT	2.93	1.26	0.39~5.46	0.30	2.32	0.025 *	1.11	
	MUST	−10.2	11.1	−32.5~12.11	−0.13	−0.919	0.363	1.23	

* *p* < 0.05. Abbreviations: Beta, standardized partial regression coefficient; CI, confidence interval; EAT-10, Eating Assessment Test; FOIS, functional oral intake scale; MMO, maximum mouth opening; MUST, malnutrition universal screening tool; NFT, number of functional teeth; SE, standard error; VIF, variance inflation factor.

## Data Availability

The author confirms that all the data generated or analyzed during this study are included in this published article; further inquiries can be directed to the corresponding author.
